# Supplementary material for: Fossil evidence unveils an early Cambrian origin for Bryozoa
Source: Nature. 2021 Oct 27;599(7884):251–5. doi: 10.1038/s41586-021-04033-w (PMC8580826; doi:10.1038/s41586-021-04033-w)
Supplement: Supplementary file 1 — This file contains Supplementary Data 1–4. (1) Derived values for box plots of Protomelission gatehousei zooid width provided in Extended Fig. 6a. (2) Raw data of zooid width and length of 13 bryozoan taxa in Extended Fig. 6b. (3) Character trait dataset and morphological states for bryozoans. (4) Character taxon matrix in NEXUS format. [file 41586_2021_4033_MOESM1_ESM.pdf]

nature portfolio

https://doi.org/10.1038/s41586-021-04033-w

Supplementary information

Fossil evidence unveils an early Cambrian origin for Bryozoa

In the format provided by the authors and unedited

Fossil evidence unveils an early Cambrian origin for Bryozoa

Zhiliang Zhang, Zhifei Zhang, Junye Ma, Paul D. Taylor, Luke C. Strotz, Sarah M. Jacquet, Christian B. Skovsted, Feiyang Chen, Jian Han & Glenn A. Brock

Supplementary Information

Content

Supplementary Data 1. Derived values for boxplots of *Protomelission gatehousei* zooid width provided in Extended Fig. 6a.

Supplementary Data 2. Raw data of zooid width and length of 13 bryozoan taxa in Extended Fig. 6b.

Supplementary Data 3. Character trait dataset and morphological states for bryozoans.

Supplementary Data 4. Character taxon matrix in NEXUS format.

Supplementary Data 1. Derived values for boxplots of *Protomelission gatehousei* zooid width provided in Extended Fig. 6a.

The colour of each series matches the colours used in Fig. 4d. Values are in µm.

		Series 1	Series 2	Series 3	Series 4	Series 5
SADME 10470<br><br>South Australia	Minimum	114	143	157	140	103
	First quartile	119	151	163	151	119
	Median	124	157	177	157	130
	Mean	123	156	175	156	136
	Third quartile	129	160	183	162	137
	Maximum	132	169	193	167	138
		Series 1	Series 2	Series 3	Series 4	Series 5
ELI XYB 4 AN02<br><br>South China	Minimum	171	200	236	214	173
	First quartile	171	206	236	216	179
	Median	186	224	239	224	199
	Mean	184	219	240	226	196
	Third quartile	194	227	245	237	211
	Maximum	194	228	247	240	215

Supplementary Data 2. Raw data of zooid width and length of 13 bryozoan taxa in Extended Fig. 6b.

Each pair of width (greatest width perpendicular to the proximal-distal axis of the zooid) and length (proximal-distal axis of the zooid) values correspond to a single zooid. N=172 biologically independent measurements of zooid size (86 zooids). For the source of measured specimens, see Extended Fig. 6b caption. Measurements are in µm.

Specimen		Width	Length
Protomelission gatehousei	SADME 10470 South Australia	120	239
		131	210
		128	204
		114	193
		143	213
		169	203
		158	204
		153	201
		158	194
		156	187
		159	215
		193	214
		184	203
		175	199
		179	188
		157	182
		163	216
		167	224
		161	200
		157	190
		156	187
		146	189
		140	168
		114	220
		131	208
		137	174
		138	199
		129	182
		121	193
		103	182
	ELI XYB 4 AN02 South China	194	278
		186	257
		171	236
		228	272
		222	253
		225	278
		200	210
		247	285

		240	255
		237	276
		236	239
		220	251
		214	254
		240	245
		228	244
		215	256
		200	256
		197	252
		173	219

**Specimen**	**Width**	**Length**
Cyclostomata		
Reptomultisparsa aff. *cricopora*	156	249
	133	285
	140	318
Microeciella suborbicularis	93	311
	100	318
	109	459
Trepostomata		
Nekhorosheviella nodulifera	210	260
	230	280
	240	280
	180	230
Orbiramus normalis	180	230
	130	190
	200	260
	170	200
Revalotrypa eugeniae	220	240
	255	300
Cryptostomata		
Prophyllodictya simplex	155	228
	179	279
	151	244
Prophyllodictya prisca	100	150
	80	140
Cheilostomata		
Pyriporopsis pohowskyi	248	393
	235	509
	288	498
Jablonskipora kidwellae	213	290
	141	200
	288	409
Acoscinopleura crassa	381	448
	395	531
Calpensia nobilis	284	763

	374	566
	355	630
Ctenostomata		
Alcyonidium condylocinereum	107	131
	83	146
	94	130
	92	117
	98	148

Supplementary Data 3. Character trait dataset and morphological states for bryozoans.

Include 21 taxa and 52 characters used to identify phylogenetic relationships shown in Fig. 4e and Extended Data Fig. 8.

No.	Character traits	States	Ref.
**1**	Overall organization	0 Solitary<br>1 Colony	1
**2**	Attachment to substrate	0 Pedicle<br>1 Cement/Encrust	1
**3**	Biomineralization	0 Absent<br>1 Present	2
**4**	Mineralogy	0 Calcareous<br>1 Apatite	2
**5**	Wall laminated	0 Absent<br>1 Present	3
**6**	Colony form	0 Bifoliate<br>1 Encrusting<br>2 Massive<br>3 Fenestrate<br>4 Ramose/Dendroid	4
**7**	Arrangement of frontal surface	0 bilaminate<br>1 unilaminate<br>2 multilaminate	5,6
**8**	Bifurcation	0 Absent<br>1 Present	5,6
**9**	Arrangement of zooid series	0 Uniserial<br>1 Multiserial	5,6
**10**	Dimensions of growth	0 Two-dimensions<br>1 Three-dimensions	5,6
**11**	Width of colony unit	0 Non-macroserial<br>1 Macroserial	5,6
**12**	Construction (proxy for flexibility)	0 Soft<br>1 Rigid	5,6
**13**	Zooid arrangement	0 Hexagonal or rhombic<br>1 Irregular<br>2 Linear	4
**14**	Mesotheca	0 Absent<br>1 Present	4
**15**	Zooid differentiation	0 Monomorphism<br>1 Polymorphism	7–9
**16**	Endozone and exozone differentiation	0 Absent<br>1 Present	3
**17**	Wall thickness at boundary between endozone and exozone	0 Absent<br>1 Present	4
**18**	Vertical wall	0 Absent<br>1 Present	2
**19**	(Zooecia) cross-section in endozone	0 Polygonal<br>1 Round	4
**20**	(Zooecia) tangential-section	0 Polygonal<br>1 Round<br>2 Rod-like	8

**21**	Frontal wall	0 Absent<br>1 Present	2
**22**	Frontal wall curvature	0 Flat<br>1 Curved	9
**23**	Frontal wall type/<br>interior wall communication	0 Fixed-walled<br>1 Free-walled<br>2 stolon	3,10
**24**	Basal wall curvature	0 Absent<br>1 Present	4,11
**25**	Frontal wall (external)<br>micro-ornamentation	0 Absent<br>1 Present	3,9
**26**	Maculae (monticules)	0 Absent<br>1 Present	5
**27**	Maculae shape	0 Irregular<br>1 Regular	4
**28**	Mesozooid	0 Absent<br>1 Present	4
**29**	Exilazooid	0 Absent<br>1 Present	4,10
**30**	Kenozooid	0 Absent<br>1 Present	3
**31**	Extrazooidal skeleton<br>(stereo)	0 Absent<br>1 Present	4
**32**	Style	0 Absent<br>1 Present	3
**33**	Style size modality	0 Unimodal<br>1 Polymodal	8
**34**	Length of autozooid	0 Short<br>1 Long (> 2 zooid chamber)	8
**35**	Zooid diaphragm	0 Absent<br>1 Present	4
**36**	Zooid diaphragm spacing	0 Close (< 1 living chamber)<br>1 Wide (> 1 living chamber)	4
**37**	Vesicular tissue	0 Absent<br>1 Present	4
**38**	Hemiphragm	0 Absent<br>1 Present	4
**39**	Medial zooid	0 Absent<br>1 Present	4
**40**	Lunaria	0 Absent<br>1 Present	8
**41**	Capitulum	0 Absent<br>1 Present	9
**42**	Cauda	0 Absent<br>1 Present	9
**43**	Median keel	0 Absent<br>1 Present	12
**44**	Zooid shape	0 Tube<br>1 Box<br>2 Vase	2,3
**45**	Pseudopores	0 Absent<br>1 Present	3

**46**	Statoblasts	0 Absent<br>1 Present	3
**47**	Pore chamber	0 Absent<br>1 Present	3
**48**	Holdfast	0 Absent<br>1 Present	1
**49**	Granular wall	0 Absent<br>1 Present	4
**50**	Budding geometry	0 Simple linear<br>1 Compound linear<br>2 Non-linear	13
**51**	Budding loci	0 Distal<br>1 Lateral+Distal<br>2 Frontal+Distal<br>3 Spiral	9,13
**52**	Regularity of budding loci	0 Regular<br>1 Irregular (more than one sites)	9,13

1. Boardman, R. S. *et al. Treatise on invertebrate paleontology. Part G. Bryozoa*. vol. 1 (Geological Society of America, Boulder, CO & University of Kansas Press, 1983).
2. Taylor, P. D., Lombardi, C. & Cocito, S. Biomineralization in bryozoans: present, past and future: Bryozoan biomineralization. *Biol. Rev.* **90**, 1118–1150 (2015).
3. Cuffey, R. J. & Blake, D. B. Cladistic analysis of the phyllum Bryozoa. *Bull. Société Sci. Nat. Ouest Fr.* 97–108 (1991).
4. Ma, J., Taylor, P. D., Xia, F. & Zhan, R. The oldest known bryozoan: *Prophyllodictya* (Cryptostomata) from the lower Tremadocian (Lower Ordovician) of Liujiachang, south-western Hubei, central China. *Palaeontology* **58**, 925–934 (2015).
5. Hageman, S. J., Bock, P. E., Bone, Y. & Mcgowran, B. Bryozoan growth habits: Classification and analysis. *J. Paleontol.* **72**, 418–436 (1998).
6. Hageman, S. J. Complexity generated by iteration of hierarchical modules in Bryozoa. *Integr. Comp. Biol.* **43**, 87–98 (2003).
7. McKinney, F. K. & Jackson, J. B. C. *Bryozoan Evolution*. (University of Chicago Press, 1989).
8. Ma, J.-Y., Buttler, C. J. & Taylor, P. D. Cladistic analysis of the 'trepostome' Suborder Esthonioporina and the systematics of Palaeozoic bryozoans. *Bryozoan Stud.* **94**, 153–161 (2014).
9. Todd, J. A. The central role of ctenostomes in bryozoan phylogeny. *Proc. 11th Int. Bryozool. Assoc. Conf.* **104**, 104–135 (2000).
10. Taylor, P. D. *Bryozoan Paleobiology*. (John Wiley & Sons, 2020).
11. Ernst, A. & Carrera, M. Upper Ordovician (Sandbian) bryozoan fauna from argentine precordillera. *J. Paleontol.* **86**, 721–752 (2012).
12. Ernst, A. *Fenestrapora* (Fenestrata, Bryozoa) from the Middle Devonian of Germany. *Paläontol. Z.* **90**, 19–32 (2016).
13. Lidgard, S. Zooid and colony growth in encrusting cheilostome bryozoans. *Palaeontology* **28**, 255–291 (1985).

Supplementary Data 4. Character taxon matrix in NEXUS format.

Character traits and all potential states are provided in Supplementary Data 3. NEXUS file is available in the Dryad Digital Repository (https://doi.org/10.5061/dryad.rn8pk0pbd).

```
#NEXUS
BEGIN DATA;
	DIMENSIONS  NTAX=21 NCHAR=52;
	FORMAT DATATYPE=standard GAP=- MISSING=? SYMBOLS = "0 1 2 3 4";
MATRIX

Eoobolus            00110--0-----0-0-0--0----0-00000--0-0000000-00010---
Phoronis            000-0--0-----0-0-0--0----0-00000--0-0000000-00010---
Protomelission      110-000011110000-0-0110100-00000-00-0000000100010000
Plumatella          110--10100011000-0-20-20-0-00000-00-0000010201000100
Alcyonidium         110--11010011010-0-20-20-0-00100-00-0000000200100211
Flustrellidra       110--40110011010-0-21-20-0-00101000-0000000200110210
Arachnidium         110--11110011010-0-20-00-0-00000-10-0000000200100201
Goryunovia          1110011100002000-1-1111000-00000-1100100100010001011
Corynotrypa         1110011100002000-1-1111000-00000-10-0000100000001011
Hallopora           1110142111101011101000-21-1010000-1100000000000011211
Orbiramus           1110142111101001010000-21-10000011110000000000000011210
Profistulipora      11100220111101000-1-00-0?-10000010110100100000011121 1
Ceramopora          1110022011100011111101-01-111100001 0-0001000000101210
Trepocryptopora     11101001111001110010 1-11-10100?1011000000000000011130
Prophyllodictya     11101001111001111010 1-11-0-00011000-1100000000011130
Nematopora          111010211110000101010-1110-00011011100000000000011130
Nekhorosheviella    11100220111010100 1-00-21-0-00001011000000000000001011
Dianulites          11100220111010100 1-00-21-0-00000011100000000000001011
Alwynopora          11100411100020010111 0-1?-0-00000-10-00000010000?1000
Fenestrapora        11101311100010 10-0-1001000-11011000-1000001000011000
Moorephylloporina   111013111000100100110-1000-00001010-0000001000011000
;
END;
```
