# Supplementary material for: Fossil evidence unveils an early Cambrian origin for Bryozoa
Source: Nature. 2021 Oct 27;599(7884):251–5. doi: 10.1038/s41586-021-04033-w (PMC8580826; doi:10.1038/s41586-021-04033-w)
Supplement: Supplementary file 2 — Reporting Summary [file 41586_2021_4033_MOESM2_ESM.pdf]

nature portfolio

Corresponding author(s): Zhiliang Zhang
Zhifei Zhang
Luke C. Strotz

Last updated by author(s): Aug 20, 2021

Reporting Summary

Nature Portfolio wishes to improve the reproducibility of the work that we publish. This form provides structure for consistency and transparency in reporting. For further information on Nature Portfolio policies, see our Editorial Policies and the Editorial Policy Checklist.

Statistics

For all statistical analyses, confirm that the following items are present in the figure legend, table legend, main text, or Methods section.

n/a	Confirmed	
☐	☒	The exact sample size (n) for each experimental group/condition, given as a discrete number and unit of measurement
☐	☒	A statement on whether measurements were taken from distinct samples or whether the same sample was measured repeatedly
☒	☐	The statistical test(s) used AND whether they are one- or two-sided<br>*Only common tests should be described solely by name; describe more complex techniques in the Methods section.*
☒	☐	A description of all covariates tested
☒	☐	A description of any assumptions or corrections, such as tests of normality and adjustment for multiple comparisons
☒	☐	A full description of the statistical parameters including central tendency (e.g. means) or other basic estimates (e.g. regression coefficient) AND variation (e.g. standard deviation) or associated estimates of uncertainty (e.g. confidence intervals)
☒	☐	For null hypothesis testing, the test statistic (e.g. F, t, r) with confidence intervals, effect sizes, degrees of freedom and P value noted<br>*Give P values as exact values whenever suitable.*
☐	☒	For Bayesian analysis, information on the choice of priors and Markov chain Monte Carlo settings
☒	☐	For hierarchical and complex designs, identification of the appropriate level for tests and full reporting of outcomes
☒	☐	Estimates of effect sizes (e.g. Cohen's d, Pearson's r), indicating how they were calculated

Our web collection on statistics for biologists contains articles on many of the points above.

Software and code

Policy information about availability of computer code

Data collection	XMReconstructor v. 7.0.2817 and ORS Dragonfly, v. 2020.2 for µCT reconstruction, visualisation and segmentation. TpsDig2 v. 2.16 for size measurement. Microsoft Excel 2016 for size data and coding phylogenetic matrices.
Data analysis	MrBayes v.3.2.7 (open source) and PAUP* v. 4.0a169 (freely available from Phylosolutions) for phylogenetic analyses. The settings needed to replicate these analyses are provided in the paper.

For manuscripts utilizing custom algorithms or software that are central to the research but not yet described in published literature, software must be made available to editors and reviewers. We strongly encourage code deposition in a community repository (e.g. GitHub). See the Nature Portfolio guidelines for submitting code & software for further information.

Data

Policy information about availability of data

All manuscripts must include a data availability statement. This statement should provide the following information, where applicable:

- Accession codes, unique identifiers, or web links for publicly available datasets
- A description of any restrictions on data availability
- For clinical datasets or third party data, please ensure that the statement adheres to our policy

All data analysed in this paper, including the phylogenetic datasets, are available as part of the Article, Extended Data Figs. 1–8, Extended Data Tables 1-2, Supplementary Information. CT scans and parameters used for scanning are available in the MorphoSource Repository (https://www.morphosource.org/concern/media/000379116 and https://www.morphosource.org/concern/media/000379121). Raw datasets are available in the Dryad Digital Repository (https://doi.org/10.5061/dryad.rn8pk0pbd).

Field-specific reporting

Please select the one below that is the best fit for your research. If you are not sure, read the appropriate sections before making your selection.

☐ Life sciences ☐ Behavioural & social sciences ☒ Ecological, evolutionary & environmental sciences

For a reference copy of the document with all sections, see nature.com/documents/nr-reporting-summary-flat.pdf

Ecological, evolutionary & environmental sciences study design

All studies must disclose on these points even when the disclosure is negative.

Study description	This is a palaeontological and taxonomic study including collection, preparation, microscopy imaging, description and phylogenetic analyses of fossil material from lower Cambrian rocks of South Australia and South China.
Research sample	The specimens used represent all currently known specimens of P. gatehousei (including the holotype) and illustrate all the key taxonomic features of this species. The are more than adequate to establish that P. gatehousei is a stem-group bryozoan
Sampling strategy	We utilise all currently known specimens of P. gatehousei (including the holotype). These specimens provide all the necessary information to establish the evolutionary affinities of P. gatehousei.
Data collection	Glenn Brock led the fossil excavation at the Ten Mile Creek section and found the holotype specimen (SADME 10470) in 1987, and Zhiliang Zhang recovered four paratype specimens (SADME 10470-1—10470-4) from acid macerated residues in 2019. Zhifei Zhang, Zhiliang Zhang and Feiyang Chen undertook fossil excavation at the Xiaoyangba section, and Zhiliang Zhang discovered ELI XYB 4 AN04. SEM, BSE and EDS images were collected using Zeiss Supra 35 VP field emission, Fei Quanta 450-FEGSEM and JEOL JSM 7100F-FESEM. µCT projections were collected using a Xradia MicroXCT-400 system. Measurements of the length, width and angle of different parts of P. gatehousei were performed on µCT and SEM images.
Timing and spatial scale	Collection of the specimens of P. gatehousei from the Ten Mile Creek section took place in 1987. Collection of the specimens from the Xiaoyangba section took place in 2015. The material collected, which represents all of the material of P. gatehousei currently known, is more than adequate to establish the evolutionary affinities of P. gatehousei.
Data exclusions	No data was excluded.
Reproducibility	This is a palaeontological study that utilises fossils that form part of the evolutionary record of this planet. They cannot be replicated or duplicated. We provide ample information in our paper for anyone to resample the localities that are the sources of these specimens and to repeat the methods of analysis we use.
Randomization	no randomization was used.
Blinding	This is a palaeontological study of all known material of a species. Blinding is inapplicable and irrelevant.

Did the study involve field work? ☒ Yes ☐ No

Field work, collection and transport

Field conditions	Lower Cambrian rocks were well-exposed and fossiliferous limestones were excavated manually in the field. The Wirrealpa Limestone has been weathered under a semi-arid climate with an average temperature of 25°C and an average amount of rainfall of 250 mm per year. The Xihaoping Member of the Dengying Formation is exposed in an area with a mild and humid climate with an average temperature of 14°C and an average amount of rainfall of 1323 mm per year.
Location	Ten Mile Creek section, Flinders Ranges, Australia: 31°15′43″ S, 138°53′15″ E<br>Xiaoyangba section, Hanzhong, China: 32°29′28″ N, 107°7′10″ E.
Access & import/export	All fossil specimens in this study were collected by the field group led by Macquarie University and Northwest University in compliance with all local, national and international laws. All collecting permissions were obtained before the collecting started. Any collecting in private land also obtained permissions from the land holder.<br><br>No permits were required to collect any of the samples included in our study.
Disturbance	All sampling was collected by hand with minimal disturbance of the surrounding environment.

Reporting for specific materials, systems and methods

We require information from authors about some types of materials, experimental systems and methods used in many studies. Here, indicate whether each material, system or method listed is relevant to your study. If you are not sure if a list item applies to your research, read the appropriate section before selecting a response.

Materials & experimental systems

n/a	Involved in the study
☒	☐ Antibodies
☒	☐ Eukaryotic cell lines
☐	☒ Palaeontology and archaeology
☒	☐ Animals and other organisms
☒	☐ Human research participants
☒	☐ Clinical data
☒	☐ Dual use research of concern

Methods

n/a	Involved in the study
☒	☐ ChIP-seq
☒	☐ Flow cytometry
☒	☐ MRI-based neuroimaging

Palaeontology and Archaeology

Specimen provenance

Specimens were collected from the lower Wirrealpa Limestone at the Ten Mile Creek section, Bunkers Graben of Flinders Ranges, Australia, and from the Xihaoping Member of the Dengying Formation, at the Xiaoyangba section of Hanzhong City, China.

No permits were required to collect these specimens

Specimen deposition

SADME 10470 and SADME 10470—110470-4 are deposited at the South Australian Geological Survey. ELI XYB 4 AN04 is deposited at Northwest University.

Dating methods

no new dates are provided in the paper.

☐ Tick this box to confirm that the raw and calibrated dates are available in the paper or in Supplementary Information.

Ethics oversight

No ethics permissions were required to undertake our study.

Note that full information on the approval of the study protocol must also be provided in the manuscript.
